# Grassland Management Affects Plant Leaf Nutrients Under Ambient and Future Climate

**DOI:** 10.1002/ece3.71615

**Published:** 2025-07-03

**Authors:** Yva Herion, Lena Philipp, Nele Detjen, Petra Hoffmann, W. Stanley Harpole, Janna Macholdt, Thomas Reitz, Christiane Roscher

**Affiliations:** ^1^ Department of Physiological Diversity Helmholtz‐Centre for Environmental Research (UFZ) Leipzig Germany; ^2^ German Centre for Integrative Biodiversity Research (iDiv) Halle‐Jena‐Leipzig Leipzig Germany; ^3^ Department of Soil Ecology Helmholtz‐Centre for Environmental Research (UFZ) Halle (Saale) Germany; ^4^ Department of Agronomy and Organic Farming Martin Luther University Halle‐Wittenberg (MLU) Halle (Saale) Germany; ^5^ Faculty of Natural Sciences 1 ‐ Biosciences Martin Luther University Halle‐Wittenberg (MLU) Halle (Saale) Germany; ^6^ Institute of Agricultural and Nutritional Sciences ‐ Crop Research Unit Martin Luther University Halle‐Wittenberg (MLU) Halle (Saale) Germany

**Keywords:** macronutrients, meadow, pasture, plant functional group, seasonal precipitation pattern

## Abstract

Climate change and agronomic management are major drivers altering Central European anthropogenic grassland ecosystems, but little is known about how these drivers interact in their effects on plant nutrient concentrations and ratios. This study was conducted in a climate change field experiment (higher temperature and changed seasonal precipitation pattern) in Central Germany with species‐rich non‐fertilized grasslands managed either by two times mowing (meadow) or three times sheep grazing (pasture) per year. In spring 2022, during peak plant growth, we collected leaves of five plant species per functional group (grasses, legumes, non‐legume forbs) as well as topsoil samples and determined plant leaf and plant available soil nutrient concentrations (N, P, K, Ca, Mg, S) and ratios. Plant functional groups differed in leaf concentrations of all studied nutrients with the exception of sulfur. The future climate treatment (compared to ambient climate) resulted in lower leaf N:P ratios across both management types and did not show any other effects on leaf or soil nutrients. Independent of the climate treatment, leaf and soil K concentrations were higher, while leaf Ca concentrations were lower in pastures compared to meadows. In addition, grasses had higher leaf N, legumes higher leaf S concentrations but lower leaf N:P ratios, and forbs lower leaf S concentrations in pastures than in meadows. While we found no interactive effect of climate and management and little effects of the rather moderate future climate treatment, the observed differences between pastures and meadows indicate that management, even at low intensity, modifies plant and soil nutrients in grasslands.

## Graphical abstract

We analyzed the effects of climate (ambient and future climate) and grassland management (meadow and pasture) on plant leaf and plant available soil nutrient concentrations (N, P, K, Ca, Mg, S) and ratios. While we found no interactive effect of climate and management and little effects of the future climate treatment, pastures and meadows clearly differed in plant leaf and soil nutrients. The responses to the treatments varied among plant functional groups.

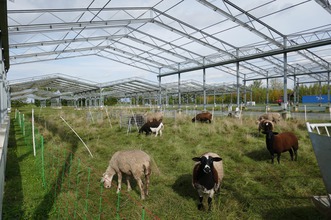

## 1 Introduction

Grasslands cover approximately 25% of the global terrestrial surface (FAO 2022) and provide multiple ecosystem functions and services (Bengtsson et al. 2019; Schils et al. 2022). While their major biomass proportion often consists of grasses, dicotyledonous forb species (legumes, non‐legume forbs) contribute to their high species diversity (Wilson et al. 2012). Grassland diversity and functioning are threatened by global change drivers such as land‐use and climate change (Gibson and Newman 2019; Macholdt et al. 2023; Schils et al. 2022). Plant tissue nutrient concentrations and ratios (i.e., stoichiometry) are key for plant growth and important for many ecosystem processes such as productivity, nutrient cycling, and trophic interactions (Aerts and Chapin 1999; Sterner and Elser 2002). The elemental composition of plants is partly phylogenetically constrained, but it is also influenced by many environmental factors such as soil fertility, climatic factors, and organismic interactions (Kerkhoff et al. 2006; Sterner and Elser 2002) and can therefore be expected to respond sensitively to global change (Sardans et al. 2021; Wang et al. 2019). The ability of the soil to provide plants with nutrients depends on the parent rock, chemical, and physical properties of the soil as well as soil biota, which decompose dead plant material and release nutrients back into the soil (Chapin et al. 2011), a process that is strongly influenced by climate factors (Castro et al. 2010; Manzoni et al. 2012; Pugnaire et al. 2019). Accordingly, in addition to direct effects, global change may influence plant nutrition indirectly through its effects on plant available soil nutrients. Studies on the impacts of global change drivers on plant nutrition have so far mainly focused on nitrogen (N) and phosphorus (P) (Kayser and Isselstein 2005; Sardans and Peñuelas 2015). However, other macronutrients, such as potassium (K), calcium (Ca), magnesium (Mg), and sulfur (S), are also involved in important plant functions, for example, osmoregulation, signal transduction, cell wall construction, light absorbance, and enzyme activation (Schulze et al. 2019) and therefore should be included in studies on global change effects (Kayser and Isselstein 2005; Sardans et al. 2021; Sardans and Peñuelas 2015).

Globally, a further increase in mean annual temperature is predicted, while precipitation changes are region‐specific (IPCC 2023). For Central Europe, climatic models predict little shifts in the annual amount of precipitation but a changed distribution over the year, with strongly decreasing precipitation amounts in summer and slightly increasing precipitation amounts in spring and fall (e.g., Jacob et al. 2014). While drought effects on plant nutrient concentrations and stoichiometry have been investigated in several studies (e.g., He and Dijkstra 2014; Luo et al. 2018; van Sundert et al. 2020), less is known about the effects of seasonally higher amounts of precipitation on plant nutrition (Dellar et al. 2018; Dumont et al. 2015). Higher water availability (especially in combination with warming) can enhance soil microbial and enzymatic activity and thereby improve plant N, P, and S availability (Lambers and Oliveira 2019; Matías et al. 2011; Stark and Firestone 1995). However, it may also promote the growth of plants with higher nutrient demands (e.g., Duffková 2017; Guiz et al. 2018; Welker et al. 2005). Consequently, meta‐analyses came to contrasting results ‐ higher water availability resulted either in higher (Dellar et al. 2018) or lower plant N concentrations (Dumont et al. 2015). If a wetter period follows a dry period, the lower nutrient uptake during the dry period can be compensated for during the wetter period (Arfin Khan et al. 2016; He and Dijkstra 2014; van Sundert et al. 2020). Immediately after rewetting, a pulse in soil nutrient availability due to, for example, increased microbial activity can lead to higher plant nutrient concentrations, which, however, are found to return to previous levels within a few months (Mackie et al. 2019; Schärer et al. 2023; van Sundert et al. 2020). The question is whether soil and plant nutrient concentrations also return to previous levels during a prolonged wetter period following a drought or whether they remain at higher levels when more water is available.

Central European grasslands have been created by human activities and require regular grazing or mowing to maintain them (Hejcman et al. 2013). In meadows, the majority of plant biomass is non‐selectively removed by mowing and only stubbles remain (Liu et al. 2023). In pastures, selective foraging by grazing animals may remove less biomass (Sanaullah et al. 2010) and large proportions of the nutrients taken up by grazing animals are non‐homogeneously returned to the soil via their excreta (Early et al. 1998; Gilmullina et al. 2020). Nitrogen, K, and S are excreted via urine directly in plant available forms (ionic K, sulfate) or are converted in the soil within a few days (urea and other N‐containing components to ammonium and nitrate), while P, Ca, and Mg are mainly excreted via dung and released slower (e.g., Bristow et al. 1992; Haynes and Williams 1993; Nguyen and Goh 1994; Saunders 1984). High soil K and nitrate concentrations after a urination event can lead to leaching of Ca and Mg (e.g., Early et al. 1998; Hogg 1981). Studies showed that grassland management can modulate climate change effects (e.g., Bazzichetto et al. 2024; Korell et al. 2024), but so far only in a few experiments including climate change and grassland management treatments, plant nutrient responses have been analyzed (Berauer et al. 2021; Deléglise et al. 2015; van Sundert et al. 2020). Previous studies on plant biomass have shown that higher management intensity and lower plant cover increase the drought sensitivity of grasslands (e.g., Bazzichetto et al. 2024; Deléglise et al. 2015; Korell et al. 2024). Accordingly, more nutrients and dead plant material may accumulate in the soil of more intensively used grasslands during drought, leading to a stronger pulse of nutrient availability after rewetting than in less intensively used grasslands, but it can be expected that this is only temporary and that the difference disappears within a prolonged wetter period (Mackie et al. 2019; Schärer et al. 2023; van Sundert et al. 2020).

A common approach to handle the great variety of grassland species in ecological studies is their classification into functional groups such as grasses, legumes, and non‐legume forbs. Plant functional groups have been found to differ inherently in nutrient concentrations due to different nutrient acquisition traits and different nutrient requirements (e.g., Furey and Tilman 2023; Zhou et al. 2024). Legumes are able to fix atmospheric N_2_ in symbiosis with rhizobia (Beijerinck 1888; Hellriegel and Wilfarth 1888) and usually have higher leaf N concentrations than other plant functional groups (e.g., Adams et al. 2016; Mathesius 2022; Vergutz et al. 2012). Grasses are often found to have lower shoot Ca and Mg concentrations compared to other plant functional groups, probably due to their lower root cation exchange capacity and their higher silicon uptake (e.g., Broadley et al. 2003, 2004). Furthermore, a recent study discussed that the nutrient uptake of grasses may generally be lower (Zhou et al. 2024). Apart from inherent differences, some studies have shown that plant functional groups can respond differently to climate change and grassland management (e.g., Dellar et al. 2018; Duffková 2017; Wang et al. 2019), probably due to their different root characteristics ‐ grasses generally have thinner roots, a denser and shallower rooting system, and lower carboxylate exudations than forbs (Zhou et al. 2024). Accordingly, forbs have been found to be more resistant to drought, while grasses recovered faster (Mackie et al. 2019; van Sundert et al. 2021). However, previous studies on the responses of plant functional groups to climate change and grassland management mostly focused on plant biomass or single nutrients such as N and P.

In this study, we analyzed plant leaf nutrient concentrations (N, P, K, Ca, Mg, S) and ratios (N:P, N:K, P:K) of 15 grassland species representing three functional groups (grasses, legumes, non‐legume forbs) as well as plant available nutrient concentrations in the topsoil (N, P, K, Ca, Mg) in a long‐term climate change field experiment comprising pastures and meadows as grassland management types (Global Change Experimental Facility (GCEF), Schädler et al. 2019). The future climate treatment includes higher mean temperatures as well as higher amounts of precipitation in spring and fall and lower amounts of precipitation in summer following regional predictions of climate change. Sampling took place in spring 2022, during peak growth of the grasslands and accordingly their highest nutrient demands. We harvested young but fully expanded leaves to exclude differences in tissue nutrient concentrations due to different proportions of plant organs and different developmental stages (Guiz et al. 2018). Before testing our hypotheses on the effects of climate change and grassland management, we checked whether plant functional groups showed inherent differences in leaf nutrient concentrations and ratios. For example, we expected legumes to have higher leaf N concentrations and grasses to have lower leaf Ca and Mg concentrations than the other plant functional groups. Our hypotheses are that (1) leaf N, P, and S concentrations are higher under the warmer and wetter conditions of the future climate treatment in spring, especially in the fast recovering grasses, because we no longer expect lagged effects of the previous summer drought, (2) leaf N, K, and S concentrations are higher in pastures than in meadows, especially in the grasses with their fine and dense rooting systems, while leaf Ca and Mg concentrations are lower in pastures than in meadows, because we expect stronger effects of urine than dung and (3) there are no interactive effects of climate and grassland management. In our experiment, mowing removes more aboveground biomass than grazing and therefore meadows may respond more sensitive to the lower amount of summer precipitation under future climate treatment, possibly resulting in a higher peak in nutrient availability after rewetting in fall. However, we assume that the difference disappears until the following spring and further, we do not expect different responses of meadows and pastures to the warmer and wetter conditions of the future climate treatment in spring.

## 2 Materials and Methods

### 2.1 Global Change Experimental Facility (GCEF)

The GCEF is located at the field research station of the Helmholtz‐Centre for Environmental Research (UFZ) in Bad Lauchstädt, Saxony‐Anhalt, Germany (51°23′33″ N 11°52′59″ E, 118 m a.s.l). The climate of the region is sub‐continental with a mean annual precipitation of 486 mm and a mean annual temperature of 10.1°C (1993–2022; Gründling et al. 2022). For spring (March to May), the long‐term mean of precipitation was 112 mm and the long‐term mean of temperature was 9.5°C. In the study year (2022), the precipitation from March to May amounted to only 62 mm, while the temperature (9.4°C) was close to the long‐term mean. The soil of the experimental site is a Haplic Chernozem with a humus‐rich topsoil horizon of more than 40 cm, a neutral soil reaction, and a high calcium carbonate content (Altermann et al. 2005, Table 1). Before the establishment of the GCEF in 2012, the site was used for crop cultivation. The experiment was set up according to a two‐factorial split‐plot design. It consists of ten main plots (80 × 24 m): five exposed to future climate and five to ambient climate as control. The main plots are divided into five subplots (each 16 × 24 m), to which five land‐use treatments were randomly assigned (conventional farming, organic farming, intensively used meadow, extensively used meadow and extensively used pasture) resulting in five replicates per climate × land‐use combination.

**TABLE 1:** Topsoil (0–15 cm depth) pH values and plant available nutrient concentrations (means and standard deviations (±1 SD), *N* = 20 (one sample per subplot)).

	Mean	SD
pH	6.63	0.53
N_min_ (mg kg^−1^ DW)	2.87	1.03
P_DL_ (mg kg^−1^ DW)	67.24	34.53
K_DL_ (mg kg^−1^ DW)	76.86	35.73
K_CEC_ (mg kg^−1^ DW)	173.51	34.43
Ca_CEC_ (mg kg^−1^ DW)	3422.24	849.05
Mg_CEC_ (mg kg^−1^ DW)	177.17	21.12

*Note:* pH was measured in a calcium chloride solution (0.01 *M*). Plant available soil K was determined by two methods ‐ double lactate (DL) and cation exchange capacity (CEC).

Abbreviations: DW = dry weight; min = mineral.

The future climate treatment is based on climate simulations from three different dynamic regional climate models with four emission scenarios for the period 2070–2100 (see Schädler et al. 2019 and references therein) predicting a temperature increase of 2°C across all seasons and a changed seasonal precipitation pattern with 10% increase in spring (March–May) and fall (September–November) and 20% decrease in summer (June–August). The climate manipulation started in spring 2014. The house‐shaped open steel constructions covering the future climate plots are equipped with movable translucent plastic tarpaulins and irrigation systems (for more details see Schädler et al. 2019). The passive night‐warming is achieved by closing the roofs and the long sides (east and west) every day from sunset to sunrise, resulting in an increase of daily mean air temperature of around 0.55°C at 5 cm above the ground (Schädler et al. 2019). In spring and fall, stored rainwater is added via the irrigation systems to increase ambient precipitation by 10%. In summer, the roofs and long sides are also closed in case of rain events during the day to reduce ambient precipitation by about 20% in the future climate treatment. Continuous monitoring of the amount of precipitation ensures that the predicted future climate is relatively accurately achieved.

In this study, we focus on the extensively managed grasslands, either used as meadows or pastures. For their establishment in 2014, seeds of 56 native grassland species (14 grasses, 10 legumes, 32 non‐legume forbs (hereafter forbs)) derived from the regional species pool were sown (for more details see Schädler et al. 2019). Starting in 2015, the meadows were mown twice a year (June, August). The grazing frequency by sheep was gradually increased, from one time in 2015 (August), to two times in 2016 (June, August) up to the intended grazing frequency of three times starting in 2017 (May, June, August). Exceptions were made in single years when mowing and grazing could not take place as planned in specific seasons due to insufficient plant growth. In the year of sampling (2022), the first grazing at the beginning of May had to be suspended because spring growth started late. Grazing is carried out as a short‐term rotational grazing with a herd of 10–13 adult sheep and 10–20 lambs remaining on each pasture subplot (16 × 24 m) for 24 h. Assuming livestock units (LSU) of 0.1 for adult sheep and 0.05 for lambs (BMEL 2025), the stocking density during grazing ranged from 39.06 to 59.90 LSU ha^−1^ and the annual average stocking rate was between 0.32 and 0.49 LSU ha^−1^ yr^−1^ with three grazing events per year.

### 2.2 Plant Sampling and Chemical Analyses

Plant leaves were sampled between May 19, and May 25, 2022, directly before the first management (mowing or grazing), during peak plant growth and accordingly highest plant nutrient demands in the grasslands. Five abundant plant species per functional group (grasses, legumes, forbs) were selected that occurred in at least three of the five subplots per climate × grassland management combination (Table 2). On each subplot, at least three individuals (or shoots) per species were chosen and the youngest but fully expanded leaves were harvested. For legumes and forbs, the leaf blade including petiole and rachis were collected, while in case of grasses, only the blade was sampled. The harvested leaves were pooled per species and subplot, dried at 70°C for 48 h and milled using a ball mill (MM200, Retsch, Germany). Approx. 10 mg of the finely milled leaf material was filled in tin capsules and measured with an elemental analyzer (Vario EL Cube, Elementar Analysensysteme GmbH, Germany) to determine N concentrations. For the determination of P, K, Ca, Mg and S concentrations, approx. 250 mg of the milled plant material was digested with 65% nitric acid and 30% hydrogen peroxide. After a pre‐digestion with 5 mL nitric acid (at least four hours), 0.5 mL hydrogen peroxide was added, the digestion vessels were closed and placed in a microwave (Mars 6, CEM GmbH, Germany) for one hour, up to 200°C. The next day, dissolved nitrous gases were removed by again adding hydrogen peroxide (0.25 mL) and afterwards the samples were centrifuged (Heraeus Megafuge 16R, Thermo Fisher Scientific, USA: 4500 × *g*, 10 min). The nutrient concentrations were measured by inductively coupled plasma optical emission spectroscopy (iCAP 7400 ICP‐OES Analyzer Duo, Thermo Fisher Scientific, USA). The resulting nutrient concentrations are given in milligram per gram dry weight (mg g^−1^ DW). Moreover, mass ratios of N to P (N:P), N to K (N:K), and P to K (P:K) were calculated.

**TABLE 2:** Plant species, their assignments to functional groups, and the number of subplots per climate × grassland management combination on which the species were sampled.

Species name	Family	Functional group	Sampled subplots per treatment combination
*Arrhenatherum elatius* (L.) P.Beauv. ex J.Presl & C.Presl.	Poaceae	Grass	3
*Bromus erectus* Huds.	Poaceae	Grass	5
*Dactylis glomerata* L.	Poaceae	Grass	5
*Festuca rupicola* Heuff.	Poaceae	Grass	3
*Trisetum flavescens* (L.) P.Beauv.	Poaceae	Grass	3
*Falcaria vulgaris* Bernh.	Apiaceae	Forb	3
*Achillea millefolium* L.	Asteraceae	Forb	5
*Tragopogon orientalis* L.	Asteraceae	Forb	3
*Scabiosa ochroleuca* L.	Caprifoliaceae	Forb	3
*Galium album* Mill.	Rubiaceae	Forb	5
*Lotus corniculatus* L.	Fabaceae	Legume	3
*Medicago falcata* L.	Fabaceae	Legume	5
*Securigera varia* (L.) Lassen	Fabaceae	Legume	5
*Trifolium pratense* L.	Fabaceae	Legume	3
*Vicia angustifolia* L.	Fabaceae	Legume	3 (2)

*Note:* *Vicia angustifolia* could only be sampled on two pasture subplots per climate treatment.

### 2.3 Soil Sampling and Chemical Analyses

Samples of the topsoil (0–15 cm depth) were taken between May 9, and May 12, 2022. On all five subplots per climate × grassland management combination, three soil cores with a diameter of 1.5 cm were taken and pooled. The soil samples were sieved to 2 mm, coarse roots and litter removed and they were stored at −20°C until further analyses. Soil mineral N was extracted from 5 g soil with 20 mL of a potassium chloride solution (1 *M*) while shaking for 1.5 h. After filtration, the concentrations of ${\mathrm{NH}}_{4}^{+}$‐N and ${\mathrm{NO}}_{3}^{-}$‐N were measured using a flow injection analyzer (FIAstar 5000, Foss GmbH, Germany). Plant available P and K were extracted from 1 g soil by adding 50 mL of a calcium lactate solution adjusted to pH 3.6 and shaking for 1.5 h. Phosphorus concentrations were measured photometrically at 900 nm (Infinite 200 PRO, Tecan Group Ltd., Switzerland) after staining molybdenum blue. Potassium concentrations were determined using an ion‐selective electrode (SevenExcellence pH/Ion meter, Mettler‐Toledo GmbH, Germany). For the extraction of exchangeable cations (K, Ca and Mg), an ammonium acetate solution (1 *M*) with a pH value (pH 7) similar to that of the soil samples (Table 1) was used to reduce errors due to the dissolution of carbonates (Carter and Gregorich 2007). The extraction solution (100 mL) was added to 2.5 g soil. The suspensions were manually shaken for 15 s, left standing for five hours, shaken for 15 s, left standing for 18 h and again shaken for 15 s (personal communication with Kuhlmann, I. & Mund, M., Max Planck Institute for Biogeochemistry, Jena). After centrifugation (Heraeus Megafuge 16R, Thermo Fisher Scientific, USA: 2900 × *g*, 15 min), the extracts were filtered and K, Ca and Mg concentrations were measured by inductively coupled plasma optical emission spectroscopy (iCAP 7400 ICP‐OES Analyzer Duo, Thermo Fisher Scientific, USA). The water contents of the soil samples were determined gravimetrically (drying at 105°C for 24 h), and plant available soil nutrient concentrations are given in milligram per kilogram dry weight (mg kg^−1^ DW, Table 1).

### 2.4 Statistical Analyses

Statistical analyses were performed with R version 4.3.3 (R Core Team 2024). To assess the effects of climate (two levels: ambient and future), grassland management (two levels: meadow and pasture) and plant functional group (three levels: grass, forb, legume) as well as their two‐ and three‐way interactions on plant leaf nutrient concentrations (N, P, K, Ca, Mg, S) and ratios (N:P, N:K, P:K), linear or generalized linear mixed effects models were calculated using the “glmmTMB” function of the “glmmTMB” package (Brooks et al. 2017). Depending on the distribution of the residuals, models with normal distribution and identity link function or models with log‐normal distribution and log link function were used. To account for the experimental design and multiple measurements per subplot and species, subplot nested in main plot and species identity were included as random effects. Starting from null models with only random effects, the models were stepwise extended by adding fixed effects in the order mentioned above. The models were fitted with the maximum likelihood method and log‐likelihood ratio tests were used to test for significant model improvements after sequentially adding the fixed effects. Post hoc comparisons were run using the “emmeans” function of the “emmeans” package (Lenth 2024). For analyzing the effects of climate and grassland management as well as their two‐way interaction on plant available soil nutrient concentrations (N, P, K, Ca, Mg), the same procedure was followed, except that the random structure only consisted of main plot. For visualizations, marginal means and standard errors were derived from the calculated models using the “emmeans” function (see above).

Furthermore, leaf N, P, K, Ca, Mg, and S concentrations (log transformed) were combined in a standardized principal component analysis (PCA) using the “PCA” function of the “FactoMineR” package (Lê et al. 2008). Effects of the experimental factors were analyzed for the sample positioning (scores) on the two leading principal components (PCs) following the same procedure as above.

## 3 Results

**FIGURE 1:**
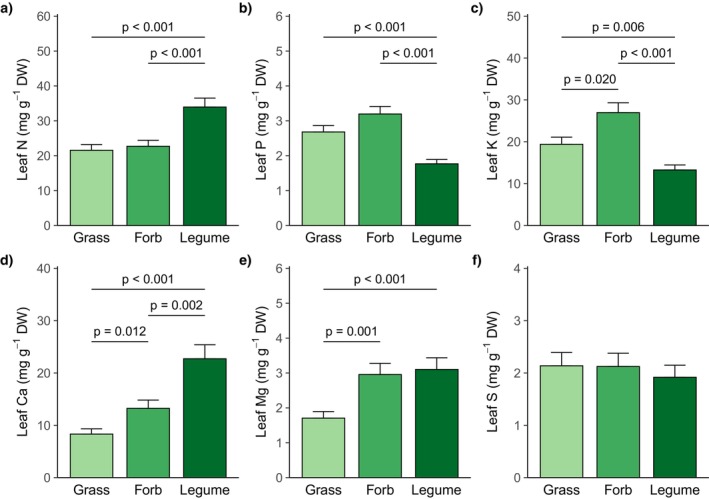
Plant leaf (a) N, (b) P, (c) K, (d) Ca, (e) Mg, and (f) S concentrations of grasses, forbs, and legumes (averaged across climate and grassland management treatments). Marginal means and their standard errors extracted from generalized linear mixed effects models (Table 3) are displayed. Statistically significant differences (*p* < 0.05, derived from post hoc comparisons) are indicated by horizontal lines and corresponding *p*‐values. DW = dry weight.

The plant functional groups differed in leaf nutrient concentrations and ratios (Table 3). Legumes had higher leaf N concentrations, but lower leaf P and leaf K concentrations compared to grasses and forbs. Leaf K concentrations also differed between grasses and forbs, with forbs having the highest leaf K concentrations (Figure 1a–c). Accordingly, leaf N:P and leaf N:K ratios were higher in legumes than in grasses and forbs, however, leaf P:K ratios were not different among plant functional groups (Figure S1a–c). Grasses had lower leaf Ca and leaf Mg concentrations than forbs and legumes. Legumes had the highest leaf Ca concentrations, which also significantly differed from those of forbs (Figure 1d,e). The plant functional groups did not differ in leaf S concentrations (Figure 1f).

**TABLE 3:** Effects of climate (CL), grassland management (MGT), and plant functional group (FG) as well as their two‐ and three‐way interactions on leaf nutrient concentrations, ratios, and sample positioning (scores) on the first two principal components (PC 1 and PC 2) of a standardized principal component analysis (PCA) combining leaf nutrient concentrations.

	df	N (mg g^−1^ DW)		P (mg g^−1^ DW)		K (mg g^−1^ DW)		Ca (mg g^−1^ DW)		Mg (mg g^−1^ DW)		S (mg g^−1^ DW)	
		*χ* ^2^	*p*	*χ* ^2^	*p*	*χ* ^2^	*p*	*χ* ^2^	*p*	*χ* ^2^	*p*	*χ* ^2^	*p*
CL	1	0.0	0.943	1.4	0.229	1.1	0.299	0.1	0.789	1.9	0.164	0.0	0.826
MGT	1	2.0	0.162	0.1	0.766	7.1	**0.008**	5.4	**0.020**	1.9	0.171	2.7	0.100
CL × MGT	1	2.7	0.103	2.4	0.119	0.1	0.764	0.9	0.336	0.0	0.946	2.9	0.090
FG	2	13.5	**0.001**	19.8	**< 0.001**	17.5	**< 0.001**	19.0	**< 0.001**	12.2	**0.002**	0.5	0.774
CL × FG	2	2.3	0.321	0.3	0.844	3.0	0.220	2.0	0.370	0.7	0.694	4.4	0.108
MGT × FG	2	8.6	**0.013**	4.7	0.094	2.5	0.280	0.8	0.658	4.4	0.109	14.8	**0.001**
CL × MGT × FG	2	0.3	0.882	0.5	0.781	4.0	0.136	0.9	0.632	2.7	0.259	2.0	0.366

	df	N:P		N:K		P:K		PC 1		PC 2	
		*χ* ^2^	*p*	*χ* ^2^	*p*	*χ* ^2^	*p*	*χ* ^2^	*p*	*χ* ^2^	*p*
CL	1	4.9	**0.026**	0.0	0.979	2.3	0.132	0.4	0.508	0.0	0.847
MGT	1	0.1	0.720	4.3	**0.038**	5.5	**0.019**	6.3	**0.012**	0.4	0.538
CL × MGT	1	0.0	0.892	0.2	0.677	1.0	0.327	0.1	0.720	1.8	0.175
FG	2	35.4	**< 0.001**	22.2	**< 0.001**	0.9	0.653	24.3	**< 0.001**	6.6	**0.037**
CL × FG	2	1.0	0.609	4.8	0.089	4.2	0.121	1.1	0.580	1.7	0.428
MGT × FG	2	7.8	**0.020**	10.5	**0.005**	2.3	0.312	0.9	0.638	13.0	**0.002**
CL × MGT × FG	2	0.1	0.972	0.6	0.745	1.7	0.427	0.3	0.845	0.0	0.992

*Note:* Results are based on generalized linear mixed effects models (N, P, K, Ca, Mg, S, and N:K) and linear mixed effects models (N:P, P:K, PC 1, and PC 2). Significant effects (*p* < 0.05) are in bold.

Abbreviations: df = degrees of freedom; DW = dry weight.

**FIGURE 2:**
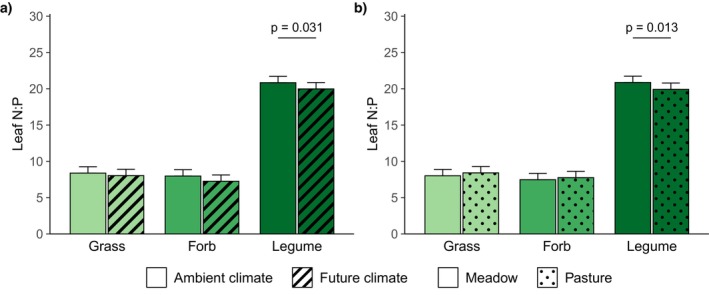
Plant leaf N:P ratios of grasses, forbs, and legumes (a) under ambient and future climate (averaged across grassland management treatments) and (b) in meadows and pastures (averaged across climate treatments). Marginal means and their standard errors extracted from a linear mixed effects model (Table 3) are displayed. Statistically significant differences (*p* < 0.05, derived from post hoc comparisons) are indicated by horizontal lines and corresponding *p*‐values.

The future climate treatment had little effects on leaf nutrient concentrations and ratios (Table 3). Leaf N:P ratios were lower under future than ambient climate across all plant functional groups and in legumes at the single functional group level (Figure 2a), while leaf N and leaf P concentrations alone did not differ significantly between the two climate treatments (Figure S2a,b). Furthermore, the future climate treatment had no effect on plant available soil nutrient concentrations (Table 4).

**TABLE 4:** Effects of climate (CL) and grassland management (MGT) as well as their two‐way interaction on plant available soil nutrient concentrations.

	df	N_min_ (mg kg^−1^ DW)		P_DL_ (mg kg^−1^ DW)		K_DL_ (mg kg^−1^ DW)		Ca_CEC_ (mg kg^−1^ DW)		Mg_CEC_ (mg kg^−1^ DW)	
		*χ* ^2^	*p*	*χ* ^2^	*p*	*χ* ^2^	*p*	*χ* ^2^	*p*	*χ* ^2^	*p*
CL	1	0.4	0.541	0.1	0.725	0.1	0.730	1.3	0.252	0.4	0.514
MGT	1	0.1	0.811	2.0	0.154	9.8	**0.002**	1.2	0.283	0.9	0.341
CL × MGT	1	0.1	0.813	1.6	0.203	0.2	0.662	0.6	0.429	0.3	0.560

*Note:* Results are based on generalized linear mixed effects models (K_DL_, Ca_CEC_, Mg_CEC_) and linear mixed effects models (N_min_, P_DL_). Significant effects (*p* < 0.05) are in bold. Model results for K_CEC_ are not shown, as these are comparable to those for K_DL_.

Abbreviations: CEC = cation exchange capacity; df = degrees of freedom; DL = double lactate; DW = dry weight; min = mineral.

**FIGURE 3:**
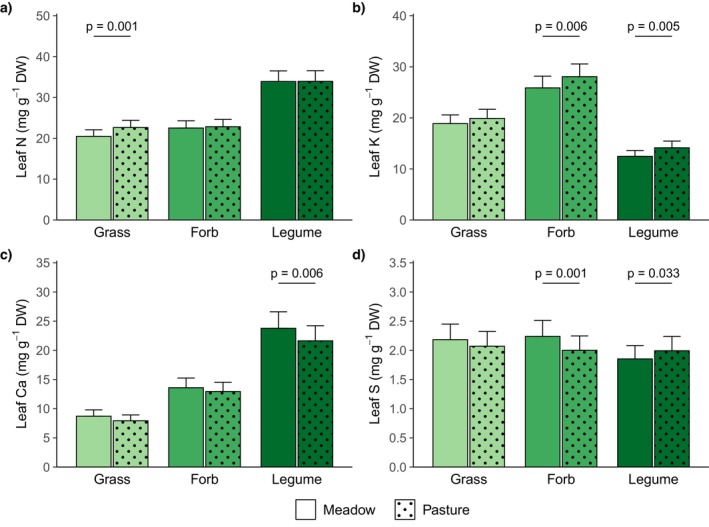
Plant leaf (a) N, (b) K, (c) Ca, and (d) S concentrations of grasses, forbs, and legumes in meadows and pastures (averaged across climate treatments). Marginal means and their standard errors extracted from generalized linear mixed effects models (Table 3) are displayed. Statistically significant differences (*p* < 0.05, derived from post hoc comparisons) are indicated by horizontal lines and corresponding *p*‐values. DW = dry weight.

**FIGURE 4:**
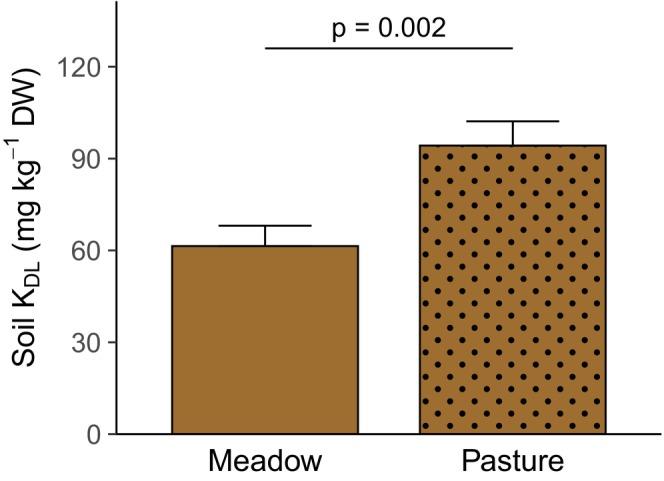
Plant available soil K concentrations (DL = double lactate) in meadows and pastures (averaged across climate treatments). Marginal means and their standard errors extracted from a generalized linear mixed effects model (Table 4) are displayed. The statistically significant difference (*p* < 0.05) is indicated by a horizontal line and the corresponding *p*‐value. Similar findings hold true for soil K concentrations determined via cation exchange capacity method. DW = dry weight.

Grassland management affected leaf concentrations and ratios of several nutrients (Table 3). Grasses had higher leaf N concentrations in pastures than in meadows, while forbs and legumes did not differ in leaf N concentrations dependent on grassland management (Figure 3a). Leaf P concentrations were similar in both management types, however, legumes had lower leaf N:P ratios in pastures compared to meadows (Figure 2b). Across all plant functional groups, leaf K concentrations were higher in pastures than in meadows, but at the level of single functional groups, the difference was not significant for grasses (Figure 3b). Consistent with the higher leaf K concentrations, leaf N:K and leaf P:K ratios were generally lower in pastures compared to meadows. At the level of single functional groups, legumes showed a significant response to grassland management in both N:K and P:K ratios, while forbs only differed in P:K ratios (Figure S3a,b). Leaf Ca concentrations were lower in pastures than in meadows across all plant functional groups and in legumes at the single functional group level (Figure 3c), while leaf Mg concentrations were similar in both grassland types. Finally, forbs had lower leaf S concentrations in pastures than in meadows, while the opposite was the case for legumes and leaf S concentrations of grasses did not differ between management types (Figure 3d). Besides, plant available soil nutrient concentrations were also affected by grassland management (Table 4). Plant available soil K concentrations (determined by double lactate or cation exchange capacity method) were higher in pastures than meadows (Figure 4). Climate and management treatments did neither interact in their effects on leaf nutrient concentrations and ratios (Table 3) nor on plant available soil nutrient concentrations (Table 4, Figure S4).

**FIGURE 5:**
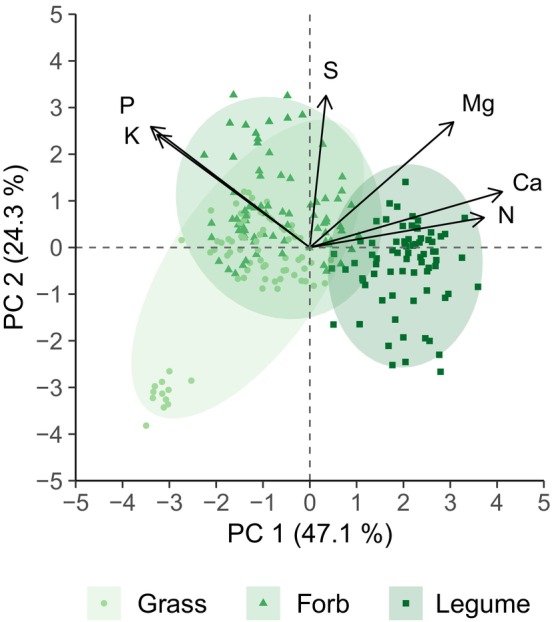
Loadings as well as sample positioning (scores) for the two leading principal components (PC 1 and PC 2) of a standardized principal component analysis (PCA) combining leaf N, P, K, Ca, Mg, and S concentrations. Ellipses indicate 95% confidence intervals for the means of plant functional groups.

In the PCA, the two leading PCs explained 47.1% and 24.3% of the total variance, respectively. The first PC had high positive loadings for leaf Ca, N, and Mg concentrations, while leaf P and K were negatively associated (Table S1, Figure 5). Along the first PC, legumes were clearly separated from grasses (*p* < 0.001) and forbs (*p* < 0.001) and pastures had lower scores than meadows across all plant functional groups (Table 3). The second PC had high positive loadings for leaf S, Mg, P, and K concentrations (Table S1, Figure 5). Along the second PC, forbs were differentiated from grasses (*p* = 0.028) and legumes (*p* = 0.047) and post hoc tests of the interaction between management type and plant functional group showed that legumes had higher scores (*p* = 0.001) in pastures than in meadows (Table 3). The future climate treatment had no effect on the sample positioning on both PCs (Table 3).

## 4 Discussion

### 4.1 Plant Functional Groups

In accordance with previous studies, our study confirms that major functional groups of herbaceous species show distinct leaf concentrations in several nutrients. Separate and multivariate analyses showed that legumes had higher leaf N and Ca concentrations, but lower leaf P and K concentrations compared to non‐legume functional groups. It has often been shown that legumes have higher leaf N concentrations than other functional groups (e.g., Adams et al. 2016; Mathesius 2022; Roscher et al. 2018; Vergutz et al. 2012) and that they accumulate Ca (Schaller et al. 2016). However, the lower leaf P and K concentrations contrast with the generally higher P and K requirements of legumes compared to species relying only on soil N (Divito and Sadras 2014). A possible explanation could be that P and K mainly accumulated in the nodules (Divito and Sadras 2014; Schulze et al. 2006) and accordingly also other studies found partly lower aboveground tissue K concentrations in N_2_‐fixing compared to non‐N_2_‐fixing species (Furey and Tilman 2023; Vergutz et al. 2012). Furthermore, in our study, forbs had the highest leaf K concentrations (cf. Furey and Tilman 2023) and grasses had lower leaf Ca and Mg concentrations than forbs and legumes, which is in accordance with our expectation and other studies before (e.g., Furey and Tilman 2023; Vergutz et al. 2012; Zhou et al. 2024). However, N, P, K, and S did not show the lowest leaf concentrations in grasses. Therefore, the recent assumption that grasses generally have lower nutrient concentrations than forbs and legumes (Zhou et al. 2024) is not supported in our study.

### 4.2 Future Climate (Hypothesis 1)

In contrast to our first hypothesis, leaf nutrient concentrations did not show any response to the future climate treatment. An increase in growth could have diluted the nutrients absorbed (e.g., Duffková 2017; Guiz et al. 2018; Welker et al. 2005). Alternatively, the drier conditions in the previous summer under future climate could have led to lower nutrient uptake, which had to be compensated for during the following wetter fall and spring resulting in nutrient concentrations similar to those under ambient climate (He and Dijkstra 2014). However, neither spring biomass production (Korell et al. 2024) nor plant available soil nutrient concentrations (Table 4) were higher under future compared to ambient climate and studies on soil microbial activity in the same experiment found no or only little effects of the future climate treatment (Kostin et al. 2021; Siebert et al. 2019; Sünnemann et al. 2021). This might be due to the rather moderate but realistic future climate treatment in our experiment, while more extreme changes in temperature or precipitation were shown to lead to altered plant nutrient concentrations (Dellar et al. 2018; Dumont et al. 2015).

Although we did not find direct effects of the future climate treatment on leaf N and P concentrations, leaf N:P ratios were, however, lower under future than ambient climate. Besides soil microbial and enzyme activity, soil moisture affects nutrient availability through its effect on nutrient diffusion in soil (He and Dijkstra 2014). The mobility of soil P is assumed to be more restricted by low soil moisture than that of soil N (Lambers and Oliveira 2019). Therefore, the responses of leaf N and P to water availability can differ in magnitude and direction and studies found higher plant N:P ratios under drought (He and Dijkstra 2014; Li et al. 2023; Wang et al. 2019). In contrast, the wetter conditions under our future climate treatment could have led to increased soil P mobility and uptake, while N supply remained unchanged or increased to a lesser extent resulting in lower leaf N:P ratios. In accordance, single other studies found lower leaf N:P ratios at higher water availability in trees and shrubs (Sardans et al. 2011; Sun et al. 2017), while a meta‐analysis concluded that leaf N:P ratios do not respond to higher water availability in grasslands, shrublands, and forests (Li et al. 2023). In our study, the future climate treatment effect on leaf N:P ratios was most pronounced in legumes. Due to their higher P requirement (Divito and Sadras 2014) and N_2_‐fixation, legumes might have had a greater need and a higher capacity to invest in N‐rich phosphatase enzymes (Houlton et al. 2008) to take advantage of the higher P mobility under the wetter conditions of the future climate treatment.

### 4.3 Grassland Management (Hypothesis 2)

In accordance with our second hypothesis, leaf K concentrations were higher, while leaf Ca concentrations were lower in pastures than in meadows. This was also reflected in the first axis of the PCA, where pastures had lower scores than meadows. For leaf N and S concentrations, the responses to grassland management varied depending on the plant functional group. Only in grasses, leaf N concentrations were significantly higher in pastures than in meadows and mineral soil N concentrations did not differ between grassland management types. The last grazing event before our sampling in spring was in the previous fall. As considerable amounts of urine N can be lost via ammonia volatilization, denitrification, and nitrate leaching (e.g., Haynes and Williams 1993; Thomas et al. 1988; Whitehead and Bristow 1990), we would probably have found a stronger effect of grazing on N concentrations, if our sampling had been closer to the last grazing event. However, other studies also did not find consistent responses of plant N concentrations to grazing (Duffková 2017; Liu et al. 2023; Sakadevan et al. 1993; Saunders 1984). As expected, the grasses in our study benefited more from the N input than forbs and legumes ‐ probably due to their finer and denser root systems in the upper soil layer (Zhou et al. 2024). In contrast to N, our study showed distinctly higher leaf and plant available soil K concentrations in pastures compared to meadows. Leaf and plant available soil K concentrations were positively related (*p* = 0.026). Increased plant and soil K concentrations in response to grazing or urine application have also been found in other studies (e.g., Duffková 2017; Sakadevan et al. 1993; Saunders 1984). Furthermore, in our study, legumes had higher, but forbs lower and grasses similar leaf S concentrations in pastures than in meadows. Sulfate leaching losses can be high especially at soil pH values around seven as in our study (Hogg 1981; Marsh et al. 1992; Nguyen and Goh 1994) and urine sulfate can be rapidly immobilized in organic forms (Williams and Haynes 1992). Accordingly, other studies also found that (even with increased soil S concentrations) no or only little urine S was taken up by plants (Sakadevan et al. 1993; Saunders 1984; Williams and Haynes 1992). The legumes in our study may have taken up more of the additional S than the other plant functional groups as they have a higher S requirement due to their N_2_‐fixation (Chapin et al. 2011). However, apart from the above‐cited New Zealand and Australian studies in intensively managed grasslands, S has rarely been included in studies on grazing effects so far.

In agreement with our hypothesis, we found lower leaf Ca concentrations in pastures than in meadows, but leaf Mg concentrations were similar. As soil Ca and Mg concentrations did not differ between grassland management types, increased Ca and Mg leaching in pastures than in meadows seems unlikely. Possibly, the lower leaf Ca concentrations in pastures than in meadows were due to the higher soil K concentrations suppressing the Ca uptake (Johansen et al. 1968; Kayser and Isselstein 2005; Sumner 2000). Other studies also reported lower plant Ca concentrations on grazed areas, even when soil Ca concentrations were higher (Duffková 2017; Saunders 1984).

Finally, in our study, legumes had lower leaf N:P ratios in pastures than in meadows, indicating higher leaf P concentrations in comparison to leaf N concentrations. In the pastures, P could have been returned via the dung of the sheep. As seen above, legumes can probably invest more in N‐rich phosphatase enzymes to benefit from additional P input. Similarly, Liu et al. (2023) also found partly lower plant N:P ratios under grazing, and several studies showed higher plant P concentrations in areas affected by dung and urine (Duffková 2017; Liu et al. 2023; Saunders 1984).

### 4.4 Interactive Effects of Climate and Grassland Management (Hypothesis 3)

In line with our third hypothesis, climate and grassland management did not interact in their effects on plant leaf nutrients and also not on soil nutrients. In our experiment, the meadows might have been more affected by the lower water availability in summer under the future climate treatment due to the more complete biomass removal with mowing compared to grazing, which might have resulted in a stronger pulse of nutrient availability after rewetting in fall. As expected, possible changes in leaf nutrient concentrations might have been of short duration, as also shown by others (Mackie et al. 2019; Schärer et al. 2023; van Sundert et al. 2020), and our pastures and meadows might not have responded differently to the higher amount of precipitation in spring under future climate treatment resulting in no interactive effects of climate and grassland management. However, additional analyses of plant and soil nutrients also including other seasons are required to support these expectations with further evidence. It is also possible that higher nutrient concentrations in our meadows ‐ whether caused by lagged effects of the previous summer or by the higher amount of precipitation in spring under the future climate treatment ‐ could have been offset by an additional nutrient input in our pastures due to the decomposition of sheep dung. The few other studies analyzing interactive effects of climate and grassland management on plant nutrition showed contrasting results (Berauer et al. 2021; Deléglise et al. 2015; van Sundert et al. 2020). Grasslands with more divergent managements (intensive vs. extensive or managed vs. abandoned) showed different responses to climate treatments (Berauer et al. 2021; van Sundert et al. 2020), while this was not the case in grasslands with more similar management types (grazing vs. mowing, Deléglise et al. 2015). Whether climate and grassland management interactively affect plant nutrients may therefore depend on how different the management types are and in which aspects they differ.

### 4.5 Conclusions

In summary, our study showed no interactive effects of the climate (ambient and future) and grassland management treatments (meadow and pasture) and generally the future climate treatment had only minor effects on plant nutrition. In contrast, the meadows and pastures showed more differences in plant leaf and soil nutrient concentrations and ratios despite the rather low grazing and mowing intensity compared to many other managed grasslands. Regarding the future climate treatment, our study followed a realistic regional scenario with a moderate increase in temperature and a changed seasonal precipitation pattern. Our sampling focused on the spring season, which is the time of peak growth in our grassland systems. However, to get a more complete understanding of how changed seasonal precipitation patterns affect plant nutrition, future studies should analyze nutrients across all seasons and over multiple years, because climate change effects on the plant–soil system may change over time.

## Author Contributions

**Yva Herion:** conceptualization (lead), formal analysis (equal), investigation (equal), writing – original draft (lead), writing – review and editing (equal). **Lena Philipp:** conceptualization (supporting), investigation (equal), writing – review and editing (equal). **Nele Detjen:** formal analysis (equal), investigation (equal), writing – review and editing (supporting). **Petra Hoffmann:** investigation (equal), writing – review and editing (supporting). **W. Stanley Harpole:** conceptualization (supporting), writing – review and editing (equal). **Janna Macholdt:** conceptualization (supporting), writing – review and editing (equal). **Thomas Reitz:** conceptualization (supporting), investigation (equal), writing – review and editing (equal). **Christiane Roscher:** conceptualization (lead), formal analysis (supporting), investigation (supporting), writing – original draft (supporting), writing – review and editing (equal).

## Conflicts of Interest

The authors declare no conflicts of interest.

## Supporting information


Data S1.

## Data Availability

This work is based on data collected within the GLIMPSE project (Global change impacts on microbiota‐plant–soil processes relevant for water and matter cycling in agricultural ecosystems) of the Helmholtz‐Centre for Environmental Research (UFZ). The datasets are openly available on Zenodo at https://doi.org/10.5281/zenodo.15516342.
