# Supplementary material for: Grassland Management Affects Plant Leaf Nutrients Under Ambient and Future Climate
Source: Ecol Evol. 2025 Jul 3;15(7):e71615. doi: 10.1002/ece3.71615 (PMC12223407; doi:10.1002/ece3.71615)
Supplement: Supplementary file 1 — Data S1. [file ECE3-15-e71615-s001.pdf]

Supplementary material

Grassland management affects plant leaf nutrients under ambient and future climate

Yva Herion[1,2], Lena Philipp[3], Nele Detjen[4], Petra Hoffmann[1,2], W. Stanley Harpole[1,2,5], Janna Macholdt[4], Thomas Reitz[2,3,6], Christiane Roscher[1,2]

[1]Helmholtz-Centre for Environmental Research (UFZ), Department of Physiological Diversity, Permoserstraße 15, 04318 Leipzig, Germany
[2]German Centre for Integrative Biodiversity Research (iDiv) Halle-Jena-Leipzig, Puschstraße 4, 04103 Leipzig, Germany
[3]Helmholtz-Centre for Environmental Research (UFZ), Department of Soil Ecology, Theodor-Lieser-Straße 4, 06120 Halle (Saale), Germany
[4]Martin Luther University Halle-Wittenberg (MLU), Department of Agronomy and Organic Farming, Betty-Heimann-Straße 5, 06120 Halle (Saale), Germany
[5]Martin Luther University Halle-Wittenberg (MLU), Faculty of Natural Sciences 1 – Biosciences, Am Kirchtor 1, 06108 Halle (Saale), Germany
[6]Martin Luther University Halle-Wittenberg (MLU), Institute of Agricultural and Nutritional Sciences - Crop Research Unit, Julius-Kühn-Straße 23, 06112 Halle (Saale), Germany

Corresponding author: Yva Herion, Helmholtz-Centre for Environmental Research (UFZ), Department of Physiological Diversity, Permoserstraße 15, 04318 Leipzig, Germany, yva.herion@ufz.de

Table S1. Explained variances and loadings for the two leading principal components (PC 1 and PC 2) of a standardized principal components analysis (PCA) combining leaf N, P, K, Ca, Mg and S concentrations.

	PC 1	PC 2
Variance (%)	47.127	24.313
N	0.792	0.136
P	-0.723	0.551
K	-0.694	0.514
Ca	0.875	0.254
Mg	0.653	0.572
S	0.074	0.693

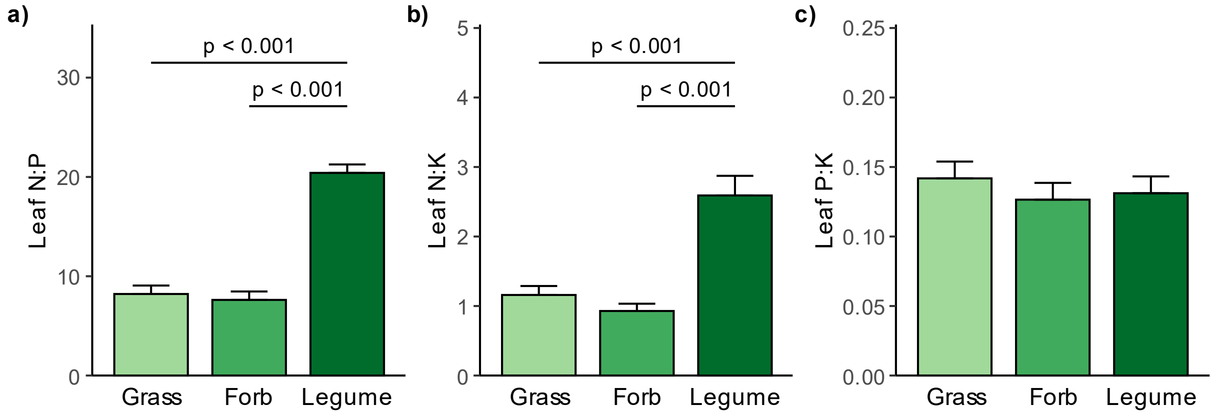

Figure S1. Plant leaf (a) N:P, (b) N:K and (c) P:K ratios of grasses, forbs and legumes (averaged across climate and grassland management treatments). Marginal means and their standard errors extracted from generalized or linear mixed effects models (Table 3) are displayed. Statistically significant differences ($p < 0.05$, derived from post hoc comparisons) are indicated by horizontal lines and corresponding p-values.

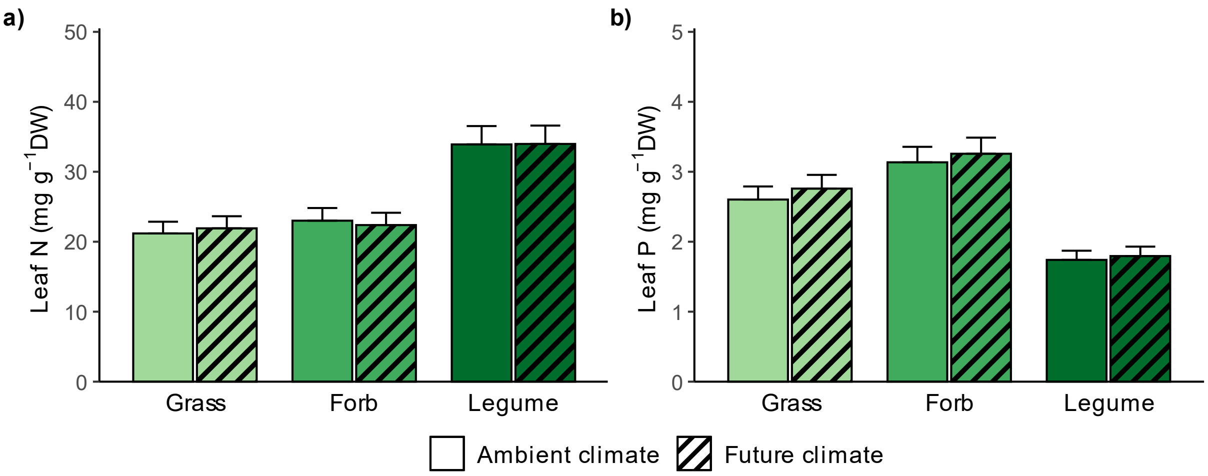

Figure S2. Plant leaf (a) N and (b) P concentrations of grasses, forbs and legumes under ambient and future climate (averaged across grassland management treatments). Marginal means and their standard errors extracted from generalized linear mixed effects models (Table 3) are displayed. There are no statistically significant differences. DW = dry weight.

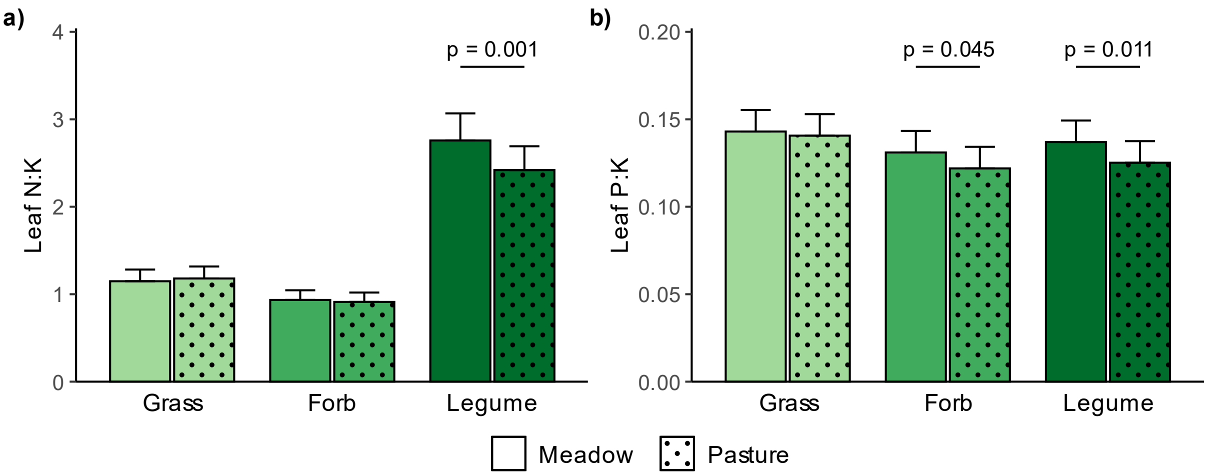

Figure S3. Plant leaf (a) N:K and (b) P:K ratios of grasses, forbs and legumes in meadows and pastures (averaged across climate treatments). Marginal means and their standard errors extracted from generalized or linear mixed effects models (Table 3) are displayed. Statistically significant differences ($p < 0.05$, derived from post hoc comparisons) are indicated by horizontal lines and corresponding p-values.

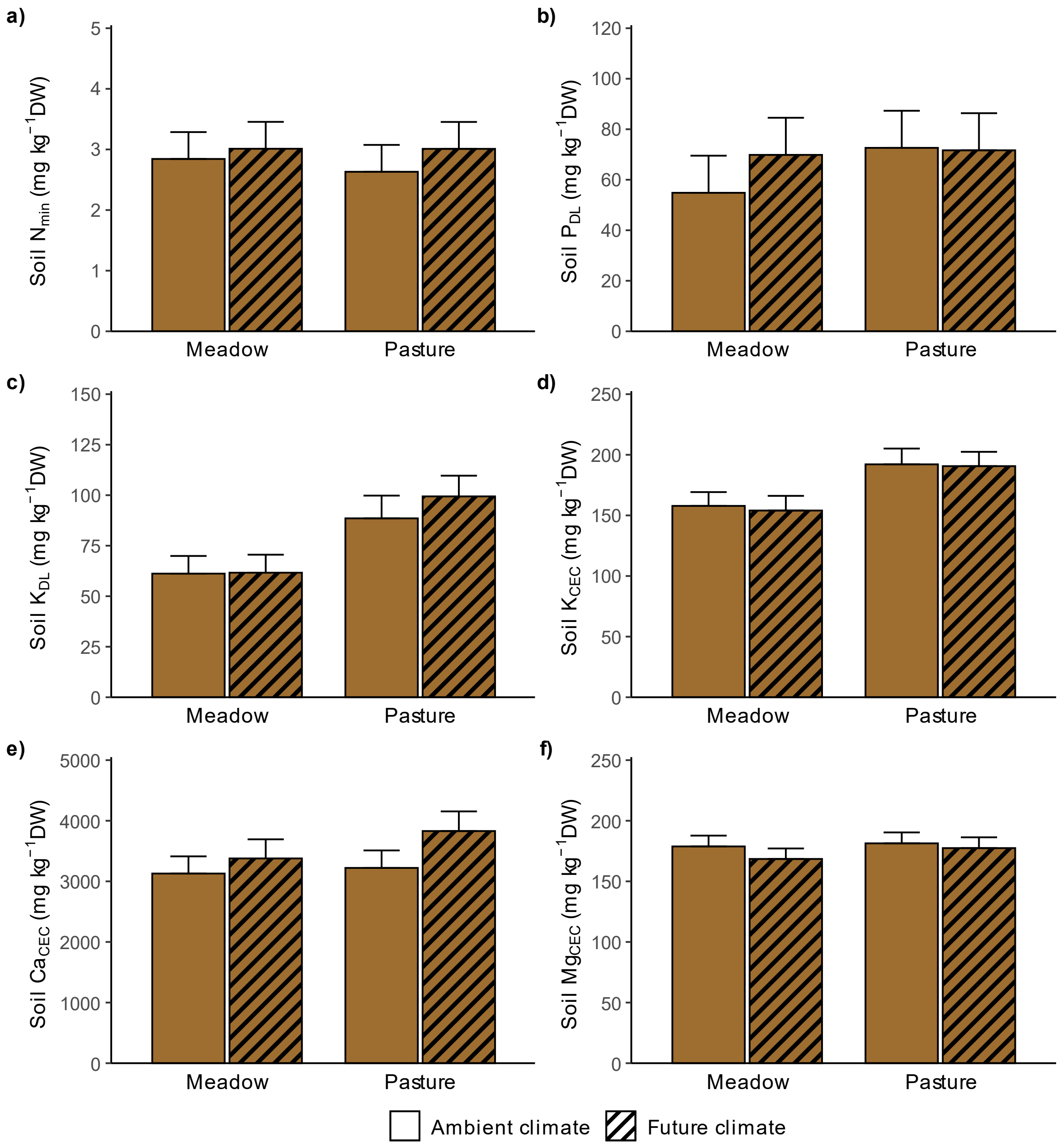

Fig. S4. Plant available soil (a) N (min = mineral), (b) P, (c) K (DL = double lactate), (d) K, (e) Ca and (f) Mg (CEC = cation exchange capacity) concentrations in meadows and pastures under ambient and future climate. Marginal means and their standard errors extracted from generalized or linear mixed effects models (Table 4) are displayed. Plant available soil K concentrations differed between grassland management types, otherwise there are no statistically significant differences. DW = dry weight.
